# Invasive Meningococcal Disease in the Vaccine Era

**DOI:** 10.3389/fped.2018.00321

**Published:** 2018-11-09

**Authors:** Simon Nadel, Nelly Ninis

**Affiliations:** ^1^Paediatric Intensive Care Unit, St. Mary's Hospital and Imperial College London, London, United Kingdom; ^2^Paediatrics, St Mary's Hospital, London, United Kingdom

**Keywords:** meningococcal, sepsis, meningitis, treatment, epidemiology, vaccine

## Abstract

Infection with the meningococcus is one of the main causes of meningitis and septicaemia worldwide. Humans are the only natural reservoir for the meningococcus which is found primarily as a commensal inhabitant in the nasopharynx in ~10% of adults, and may be found in over 25% of individuals during adolescence. Prompt recognition of meningococcal infection and early aggressive treatment are essential in order to reduce mortality, which occurs in up to 10% of those with invasive meningococcal disease (IMD). This figure may be significantly higher in those with inadequate or delayed treatment. Early administration of effective parenteral antimicrobial therapy and prompt recognition and appropriate management of the complications of IMD, including circulatory shock and raised intracranial pressure (ICP), are critical to help improve patient outcome. This review summarizes clinical features of IMD and current treatment recommendations. We will discuss the evidence for immunization and effects of vaccine strategies, particularly following implementation of effective vaccines against Group B meningococcus.

## Epidemiology of meningococcal disease

*Neisseria meningitidis* is an obligate human commensal which resides in the nasopharynx. The estimated carriage rate is estimated as between 0.6 and 34%. This figure may be higher in adolescents, young adults and in individuals living in overcrowded or confined spaces (1, 2).

There are several known serogroups of *N. meningitidis* which cause disease. The majority (more than 90%) of invasive disease is caused by just six serogroups A, B, C, W, X, and Y. The distribution of serogroups causing disease varies with age group and geographical location (1).

Approximately half a million cases of IMD occur worldwide each year, with a mortality rate of ~10% (3). Figures regarding incidence of IMD worldwide is difficult to ascertain because of inaccuracies in reporting and variations in bacteriological surveillance in different countries around the world, together with recognized under-reporting in many parts of the developing world. In the developed world, the known incidence of IMD has decreased to < 1 case per 100,000 population per year.

In the meningitis belt of sub-Saharan Africa, pandemics of meningococcal disease occur regularly and attack rates may exceed 800 cases per 100,000 population per year. In some countries in this region attack rates may be as high as 1 person in every 100 (3).

The incidence of disease caused by the different serogroups is constantly changing, both around the world and in different countries, not only because of selection pressure following introduction of effective vaccines and differences in antimicrobial usage, but also due to stochastic variations in epidemiology due to unknown reasons, including changes in population behaviors and movements of large numbers of people due to air travel.

Serogroup A meningococcus (MenA) was very common in the developed world in the early part of the twentieth Century. However, for reasons that are unclear, since the 1970s it has virtually disappeared as a cause of invasive disease and nasopharyngeal colonization in Western Europe. MenA was the most common cause of IMD worldwide, due to it being the cause of major epidemics of IMD in sub-Saharan Africa, with an incidence approaching 1 case/100 population, and associated mortality rate reaching 75% in children, adolescents and young adults. However, following the introduction of a hugely successful vaccination campaign in the meningitis belt in sub-Saharan Africa, MenA has been virtually eliminated as a cause of epidemic meningitis (4).

Serogroup B meningococcus (MenB) is the cause of endemic disease in much of the developed world, including North America, Canada, Western Europe, Australasia and South America. Following the successful introduction of vaccines which are effective against serogroup C meningococcus (MenC) in many parts of the world, MenB is now causes ~60% of IMD in the developed world. Nearly half of this disease burden occurs in children < 2 years of age (5). In the last 2 years MenB vaccine has been routinely introduced into the infant schedule in the UK and is available for university and other outbreaks in other parts of the world, including North America and Canada. Although initial reports of a reduction of disease incidence following infant immunization are encouraging, we await formal incidence data (6).

Serogroup C meningococcus (MenC) is common in the developed world and occasionally causes outbreaks and epidemics. The incidence of MenC disease has decreased in those countries which have introduced effective conjugate vaccines against MenC, including much of Western Europe and Canada (5).

In recent years there has been a dramatic increase in the number of cases of IMD caused by serogroup W (MenW). In 2016/2017, there were 225 cases of MenW disease in all ages in the UK (amounting to around a third of total reports). MenW may also contain the hypervirulent ST11 complex and has been associated with an atypical presentation of gastro-intestinal symptoms and shock without a rash. Misdiagnosis is therefore common and partly because of this, MenW is associated with a high case fatality rate (7).

Serogroup Y meningococcus (MenY) is becoming an increasingly important cause of meningococcal disease in the USA and is more recently being increasingly reported from the UK (7).

The changes in epidemiology of carriage and invasive disease of MenW and MenY has led to alterations in vaccine schedule, including introduction of MenACWY vaccine in adolescents in the UK.

Serogroup X meningococcus (MenX) is increasingly being reported in parts of sub-Saharan Africa as a cause of IMD (3).

The UK has the only national meningococcal disease vaccine programme in the World which covers serogroups A, B, C, W, and Y. However, IMD will continue to occur as the vaccine programme is not universal—i.e., not all ages are covered and no vaccine is 100% effective.

## Invasive meningococcal disease

Young children, adolescents, and young adults suffer the greatest burden of disease from the meningococcus. Children are particularly vulnerable to IMD because of their relative immune immaturity, in particular their relative under-responsiveness to pure polysaccharide antigens such as the meningococcal capsule. Over 75% of all cases of meningococcal meningitis and septicaemia occur in children < 5 years of age. Adolescents and young adults are the group with the highest prevalence of nasopharyngeal carriage, and IMD is also relatively common in this age group (8).

The World Health Organization (WHO) estimates that ~170,000 deaths occur each year from meningococcal and other bacterial meningitis and meningococcal septicaemia worldwide; the case fatality rate from invasive disease can be up to 50%, even with appropriate treatment. In addition, the estimated median risk of at least one major or minor long-term sequelae is ~20% (with a range of 12.3–35.3%). In less developed low to middle income countries, IMD remains the fourth most common cause of disability (9).

The epidemiology of bacterial meningitis and septicaemia worldwide has changed dramatically in the last 20 years following introduction of highly effective conjugate protein/polysaccharide vaccines.

Before the introduction of the conjugated vaccine against Haemophilus influenzae type b (Hib), this was the most common cause of bacterial meningitis worldwide. More recently, introduction of highly effective multivalent conjugated vaccines against *Streptococcus pneumoniae* and against MenC have resulted in significant reduction in disease burden due to these organisms.

There remain considerable problems with diagnosis, particularly in the developing world with underdeveloped microbiological services and without up to date diagnostic methods such as polymerase chain reaction (PCR).

The advent of PCR diagnostics has dramatically improved detailed microbiological diagnosis, including serogroup and subtype, for microbiological confirmation and epidemiological tracing to aid public health and outbreak control (10).

### Clinical features of meningococcal disease

It is unclear why the meningococcus invades the bloodstream after nasopharyngeal colonization to cause invasive disease. It is likely that bacterial virulence factors, environmental conditions and innate host susceptibility all play important roles.

Clinical manifestations of IMD in any individual are determined by the extent of host inflammatory factors such as activation of the immune system and host inflammatory response. These are clearly influenced by host genetic differences in constituents of the host inflammatory response regulation including those factors in the complement system, cytokines, and chemokines and the coagulation cascade. Bacterial factors, such as capsular serogroup, the amount of endotoxin released from the cell wall during growth and proliferation and bacterial load are also likely to be extremely influential in determination of the host inflammatory response (11).

In some affected individuals there may be fulminant progression of disease, followed by multi-organ failure and death within just a few hours. Even when IMD is diagnosed early and appropriate treatment is rapidly initiated, mortality rate may be up to 10%.

Of those who survive IMD, approximately 30% suffer sequelae, particularly affecting physical, cognitive, and psychological functioning. This may lead to obvious neurological impairment, nerve deafness, amputation of limbs or digits, or skin scarring (9, 12). Prompt recognition of disease and early aggressive treatment with antibiotics and fluid resuscitation are most important. Rapid identification and treatment of disease complications such as shock, raised intracranial pressure (ICP) and seizures, are vitally important to improve outcome.

### Presentation of disease

In the early stages of disease, the clinical picture is non-specific and may be confused with common trivial viral illnesses. The finding of the classical haemorrhagic rash in a child with fever is highly suggestive of IMD, However, up to 20% of children with IMD will have no rash or a non-specific maculopapular rash, and in many cases appearance of the classical haemorrhagic rash may be delayed until the disease is well advanced (13).

Non-specific clinical features such as fever, tachycardia, cold hands and feet, leg pain, and mottled or blue skin color are often present in the first 12 h of disease. The onset of the characteristic haemorrhagic petechial/purpuric rash may be delayed beyond this point, sometimes appearing up to 24 h after the first symptoms.

The classical signs and symptoms of meningism are more common in older children with meningitis. The textbook symptoms and signs of meningitis such as fever, headache, photophobia, neck stiffness, and altered mental status may not be present completely or may not be present at all, particularly in infants and younger children. A high level of suspicion of meningitis is crucial to making an early diagnosis.

### Disease progression

Invasive meningococcal disease in infants, children and young adults usually presents as two main clinical syndromes—shock and meningitis—which may exist on their own or co-exist. It should be emphasized that these two specific syndromes may overlap to a certain extent and may occur simultaneously and determine the priorities for treatment.

#### Shock

Meningococcal septic shock is characterized by a fulminant host inflammatory response to bacterial invasion. The disease may rapidly progress to cardiovascular failure, disseminated intravascular coagulopathy (DIC), multi-organ failure, and death unless rapid and aggressive resuscitation, together with organ support is initiated. Shock is present in ~20% of patients with IMD and is associated with high mortality and morbidity.

#### Meningitis

Meningitis is the most common clinical manifestation of IMD. It may develop following a longer period of low-grade bacteraemia and has a less fulminant course than shock. Patients may exhibit signs of meningeal irritation (meningism) which may progress to a depression in the level of consciousness, coma, seizures, and features of raised intracranial pressure (ICP).

Rarely, patients with meningococcal meningitis may present very acutely after a short prodromal period, with acute signs of raised ICP which rapidly progresses to deep coma, brain-stem herniation, and death.

Shock and meningitis may co-exist and present a formidable management challenge.

Invasive meningococcal disease may progress and evolve rapidly, even after appropriate treatment has been initiated. Therefore, all children admitted with suspected meningococcal disease should be closely monitored for signs of disease progression and deterioration. An improved clinical outcome critically depends on the prompt recognition of life- or limb-threatening complications including shock and/or raised ICP.

We will focus on the management of shock, raised ICP, antibiotic treatment, and adjunctive treatment modalities (14).

### Meningococcal septic shock (figure 1)

Shock is defined as inadequate delivery of oxygen and nutrients to the tissues. In IMD it results from a combination of hypovolaemia secondary to development of capillary leak syndrome due to endothelial cell dysfunction, impaired myocardial function, abnormal vasomotor tone, and impaired cellular metabolism. In a proportion of patients, relative adrenal insufficiency is present.

**Figure 1:**
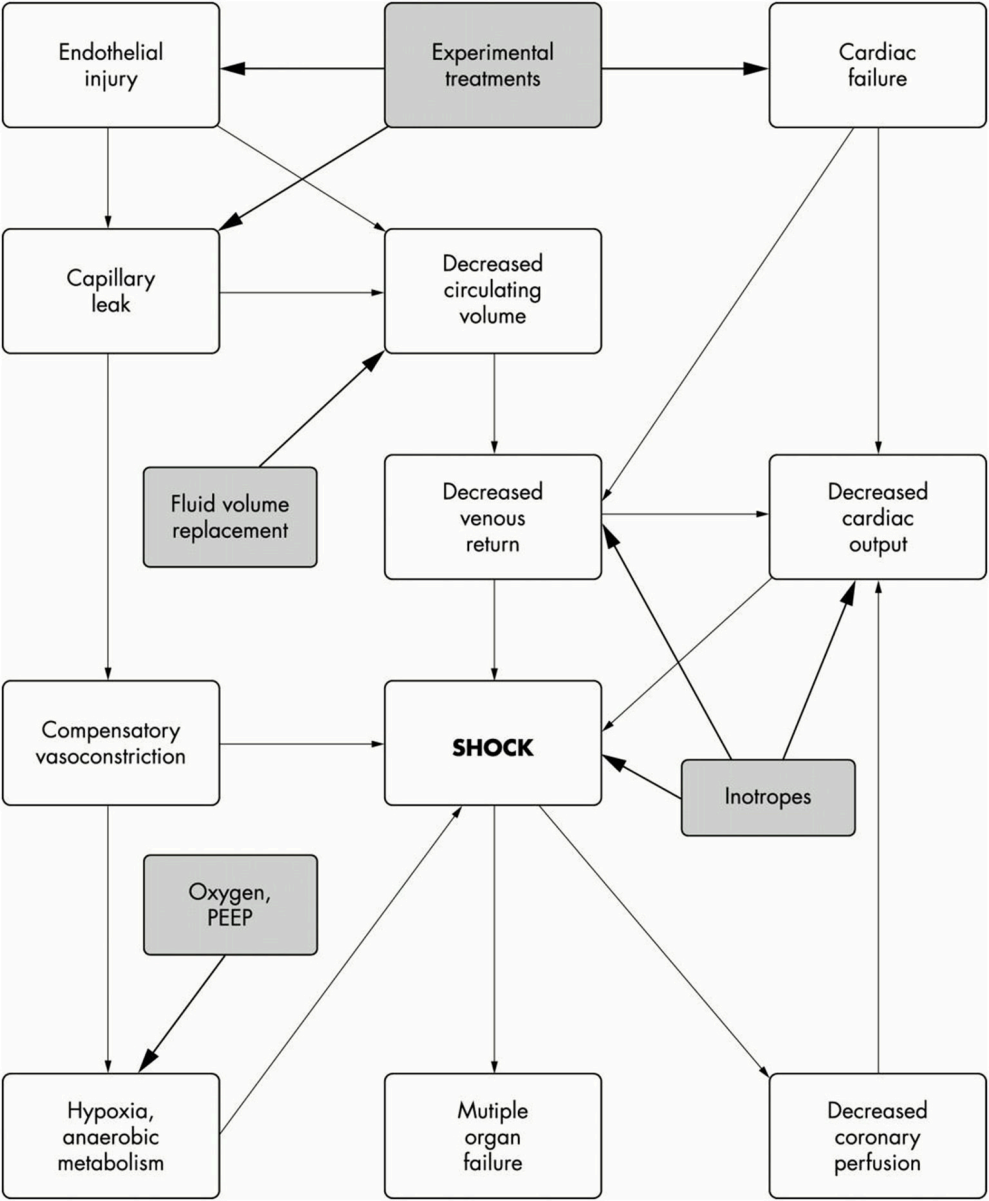
Pathophysiology of septic shock. Filled boxes are the treatment options. Taken from Welch and Nadel (15).

The clinical features of compensated shock arise because perfusion of vital organs, including brain and heart is maintained at the expense of perfusion of more expendable organs such as the skin, kidneys, and gut. In the early phases of shock this redistribution of circulating blood volume compensates for the hypovolaemia and maintains circulating blood volume and cardiac output. As a result of these compensatory mechanisms, patients with meningococcal septicaemia often present with cold hands and feet, prolonged capillary refill time and reduced urine output. As the disease progresses, ischaemia of the skin or even whole limbs may occur. In addition, many patients with shock will develop acute kidney injury, oligo/anuria, and may require renal replacement therapy.

Despite the presence of shock, cerebral perfusion and therefore function will be preserved until decompensation occurs, so that the child's relatively alert state may make even experienced clinicians underestimate the degree of cardiovascular collapse.

Development of hypotension signifies the failure of the compensatory circulatory mechanisms. We must emphasize that to diagnose shock in children, one should not rely on the demonstration of systemic arterial hypotension. Children can compensate for the loss of a significant amount of their circulating blood volume without a reduction in their blood pressure and therefore they may have normal blood pressure until shock is well-advanced.

Myocardial dysfunction is invariably present in children with meningococcal septicaemia and shock, and arises due to a number of different pathological processes.

Hypovolaemia (from capillary leak and abnormalities in vasomotor tone) leading to decreased cardiac filling; the presence of hypoxia (due to pulmonary dysfunction), acidosis and multiple electrolyte abnormalities including hypokalaemia, hypocalcaemia, hypophosphataemia, hypomagnesaemia, hypoglycaemia, and disturbed fatty acid metabolism all impair myocardial contractility; bacterial products and inflammatory mediators such as tumor necrosis factor and interleukin 1 directly suppress myocardial contractility. Interleukin 6 (IL-6) has been demonstrated to have specific myocardial depressant properties in IMD (16).

Cardiac output may improve with intravenous fluid volume resuscitation and correction of metabolic derangements. However, patients often require treatment with vasoactive agents and inotropes to improve myocardial function.

#### Initial assessment and management

Initial assessment of all patients with any potentially life-threatening illness follows the usual treatment algorithms which concentrate on management of “A, airway; B, breathing; C, circulation.”

Clinical assessment should be aimed at detecting features of shock, raised ICP, DIC and the need for advanced organ support. Assessment should be repeated frequently to evaluate response to treatment and to identify clinical deterioration.

The “A”irway is usually patent in meningococcal disease, unless consciousness is impaired. “B”reathing may be compromised by development of pulmonary oedema due to capillary leakage of fluid in the lungs, leading to hypoxia, and respiratory distress. “C”irculation is affected as described above.

#### Management of shock

There are many evidence-based treatment guidelines available for the appropriate management of children with septic shock, including that due to meningococcal disease.

Probably the most widely used are the Surviving Sepsis Guidelines, which incorporate guidelines for the management of shock in infants and children. The latest iteration of these were published in 2012 and have recently been updated (17, 18). These guidelines and more recent evidence pointing to improvement in outcome associated with complete bundle compliance suggests that implementation of treatment guidelines including a “recognition bundle” containing a trigger tool for rapid identification of patients with septic shock; a “resuscitation and stabilization bundle” to help adherence to best practice principles; and a “performance bundle” to identify and overcome perceived barriers to the pursuit of best practice principles, can lead to significant improvement in mortality and presumably morbidity (19).

The primary aim of circulatory support in shock is to maintain and replenish adequate tissue perfusion for the supply of oxygen and nutrients to the cells. To achieving this goal the clinician must adequately replenish the circulating volume with appropriate fluid resuscitation. Early and aggressive fluid resuscitation is associated with improved survival in pediatric septic shock (14). Inotropic support is often required in order to maintain adequate cardiac output and organ perfusion.

It is recommended that an initial bolus of 20 mL/kg of fluid should be given over 5–10 min to children with signs of shock. The expected response to this volume replacement is reduction in heart rate, improved peripheral perfusion, decreased capillary refill time, and improved urine output. In milder cases, shock is reversed by this initial fluid bolus, but regular repeated review is mandatory, as despite appropriate management, the disease may progress.

In patients with more advanced shock, aggressive fluid resuscitation may need to continue in order to reverse shock. In these patients, establishment of secure central venous access is a priority. This will aid and guide adequate fluid resuscitation, safe administration of vasoactive agents and measurement of central venous oxygen saturation (ScvO_2_). Central venous oxygen saturation is a useful guide to the adequacy of oxygen delivery, with the goal of achieving ScvO_2_ >70% and central venous pressure (CVP) of 8–12 mmHg (17).

If signs of shock persist after 40–60 mL/kg of fluid resuscitation given within an hour, there is a significant risk of pulmonary oedema developing with further fluid resuscitation. Elective tracheal intubation and initiation of mechanical ventilation is recommended at this stage, even without evidence of overt respiratory failure being present. If performed early, before respiratory failure is obvious, intubation and ventilation is associated with an improvement in outcome and is thought to be beneficial by allowing reduction of myocardial and respiratory muscle oxygen consumption and by facilitating delivery of positive end expiratory pressure (PEEP) to aid oxygenation (14). The sedation and muscle relaxation used in these circumstances also facilitates placement of arterial and central venous catheters.

Response to fluid resuscitation should be monitored continuously by repeated assessment of vital signs including heart rate, blood pressure, CVP, urine output, metabolic status, and peripheral perfusion. Fluid resuscitation with volumes in excess of 60 ml/kg are often needed for adequate enhancement of circulating volume. Development of pulmonary oedema and/or hepatomegaly suggest that further fluid resuscitation may not be beneficial, and may actually be harmful, as fluid overload may be present. Further support with vasoactive agents would be indicated if signs of fluid overload develop.

Controversy persists regarding the optimal fluid for resuscitation in children with septic shock. Our practice is to use 5% human albumin solution (HAS) as our preferred resuscitation fluid in septic shock of any cause. Use of 5% HAS has been associated with a reduction in morbidity and mortality in some adult studies (20). However, no equivalent studies have been performed in children with septic shock. Blood products may be required to correct anemia, thrombocytopenia, and coagulopathy, although there is controversy regarding correction of coagulation in the absence of overt bleeding.

To improve myocardial function which is invariably depressed in septic shock, treatment with adrenaline or noradrenaline should be initiated early, preferably via a central vein. It is usually impractical to gain central venous access in children before the child is sedated for tracheal intubation. Dilute dopamine or noradrenaline can be administered through a peripheral vein, or the intra-osseous route can be used to deliver concentrated solutions of adrenaline and noradrenaline until central venous access is obtained.

There is some evidence that shock refractory to inotropes may be more common in children with impaired adrenal responsiveness. Studies in adults with septic shock have documented adrenal hypo-responsiveness associated with inotrope unresponsiveness and suggested that low-dose steroid supplementation may improve survival. However, a more recent study failed to confirm these findings (21). A recently published study suggests that hydrocortisone together with fludrocortisone may confer a survival benefit in adults with septic shock (22). A meta-analysis of pediatric studies found no benefit for steroids in reducing mortality, duration of shock or length of hospital stay (23). The Surviving Sepsis campaign 2012 guidelines suggest use of low-dose replacement hydrocortisone therapy in children with fluid–refractory and catecholamine–resistant shock with suspected or proven adrenal insufficiency. It is suggested that a random cortisol level should be taken before steroids are given and a short ACTH test should be carried out at some stage to determine the need for ongoing steroid supplementation.

Most guidelines would suggest that advanced organ support for refractory shock should be implemented.

The use of renal replacement therapy or plasma exchange in sepsis has been recommended in some treatment guidelines. However, there is no evidence that these modalities improve outcomes in pediatric septic shock (24).

For children with shock refractory to increasing doses of inotropes who have severe cardio-respiratory failure, referral for Extra Corporeal Life Support (ECLS) may provide some survival benefit. However, reports of use of this modality are primarily anecdotal and ECLS is not widely available (25).

#### Respiratory support

Facial oxygen should be delivered routinely from the outset in any child with sepsis. If no major airway or breathing problem is present, priority is given to assessment and management of the circulation. The indications for immediate endotracheal intubation and mechanical ventilation are hypoxia with respiratory distress, which may indicate progression of pulmonary oedema; persistent shock despite fluid resuscitation of >40 ml/kg and need for further fluid resuscitation; fluctuating or decreasing conscious level (Glasgow Coma Score < 8, or a decrease of 3 points within 1 h); or signs of raised ICP.

Induction of anesthesia for tracheal intubation in shocked children may exacerbate cardiovascular instability. The presence of an experienced pediatric anesthetist and/or intensivist should be available. When possible, the cardiovascular system should be stabilized before intubation by adequate fluid resuscitation and initiation of vasoactive agents but the potential for acute decompensation should be anticipated.

A cuffed endotracheal tube should be used for tracheal intubation, as pulmonary oedema is likely to develop, and high ventilator pressures may be needed to maintain oxygenation and ventilation.

#### Disseminated intravascular coagulopathy (DIC)

Disseminated intravascular coagulopathy (DIC) is common in severe sepsis, regardless of the etiology. Both pro-coagulant and anticoagulant pathways are dysregulated as a consequence of activation of the inflammatory and coagulation cascades, in addition to the presence of endothelial cell dysfunction (26).

The coagulopathy that is present in meningococcal septicaemia and shock arises from a combination of the loss of the anticoagulant proteins C and S from the plasma, and the failure of anticoagulant mechanisms on the endothelial surface due to loss of surface proteins and glycosamionoglycans. The endothelial receptors which regulate protein C activation (endothelial protein C receptor and thrombomodulin) have been found to be down-regulated in patients with meningococcal septicaemia (27). Levels of circulating activated protein C and antithrombin III are reduced; the normal fibrinolytic mechanisms are suppressed due to reduced production of endothelial tissue plasminogen activator, and increased production of plasminogen activator inhibitior-1 (PAI-1) and other fibrinolysis inhibitors such as thrombin-activatable fibrinolysis inhibitor (TAFI). Together, all of these abnormalities result in uncontrolled microvascular thrombosis, suppression of degradation of intravascular thrombi, resulting in the clinical syndrome of DIC and Purpura Fulminans.

This may result in spontaneous pulmonary, gastric or cerebral hemorrhage, particularly in the presence of any associated thrombocytopenia. Correction of severe coagulopathy with vitamin K, fresh frozen plasma, platelets and, cryoprecipitate may prevent life-threatening hemorrhage. To this end regular blood cell count and coagulation studies should be monitored.

Recombinant activated Protein C (aPC) was initially shown to reduce mortality in adults with severe sepsis and septic shock. However, a study of the use of aPC in children with severe sepsis, including purpura fulminans and meningococcal disease failed to show any benefit, and possibly showed harm in younger infants with thrombocytopaenia (28). Therefore, aPC is no longer available as a therapeutic option in sepsis.

#### Biochemical derangements

Patients with septic shock often have severe derangements in blood biochemistry. These should be sought by repeated sampling and corrected when detected. Regular evaluation of blood electrolytes (including sodium which is particularly important in the context of raised ICP, potassium, phosphate, calcium, and magnesium) is important as derangements in homeostasis may impair cardiovascular status. Blood urea and creatinine should be monitored to assess renal function and need for renal replacement therapy.

Regular and repeated blood gas analysis should be carried out to identify evolving metabolic acidosis, monitor blood glucose, and electrolyte abnormalities and follow serial lactate levels. Correction of severe metabolic acidosis with Sodium Bicarbonate may result in improved cardiovascular status and improved response to inotropes.

Lactate clearance has been shown to be an important indicator of the adequacy of resuscitation. Failure to clear lactate has been shown to be a poor prognostic indicator in pediatric septic shock (29).

#### Skin and limbs

Purpura fulminans is commonly associated with meningococcal septicaemia. The skin may be severely affected in meningococcal septicaemia because of inadequate skin perfusion and microvascular thrombosis. The capillary leak may exacerbate tissue oedema and cause a compartment syndrome.

The role of fasciotomy in preserving limb viability in purpura fulminans is a subject of debate, but is indicated in circumstances where there is ischaemia in the presence of documented increased compartment pressure. Multi-disciplinary input from intensivists, orthopedic, vascular, and plastic surgeons is vital before a decision regarding surgical intervention is taken. Amputation should not be performed until it is felt to be unavoidable and only following extensive multidisciplinary discussion (30).

### Raised intracranial pressure

Raised ICP occurs due to inflammation of the meninges in meningitis and capillary leakage from cerebral blood vessels, leading to cerebral oedema. Clinically significant raised ICP is rare. Although most critically ill children with IMD have shock as their primary clinical presentation, a minority present with signs of raised ICP as their predominant clinical manifestation.

Raised ICP may restrict cerebral blood flow. To understand the physiologic impact of raised ICP on cerebral blood flow one must be familiar with the physiology of the intracranial vault.

Cerebral blood flow (CBF) supplies oxygen and nutrients to the brain. Three major factors regulate CBF: Cerebral perfusion pressure (CPP), partial pressure of arterial CO_2_, and partial pressure of arterial O_2_. Hypoxia causes cerebral vasodilation and increases CBF, and therefore ICP. Hypercapnia causes cerebral vasodilation and similarly affects CBF and ICP. Hypocapnia causes cerebral vasoconstriction, and thus reduces CBF and ICP.

CPP is the difference between mean arterial pressure (MAP) and ICP, and represents the pressure gradient across the cerebrovascular bed (CPP = MAP – ICP). In meningitis, normal cerebral vascular auto-regulation is lost, and therefore cerebral perfusion becomes critically dependent on CPP. MAP can be increased by vasopressors to increase CPP. A CPP of at least 50 mmHg has been associated with an improved outcome in traumatic brain injury, but no such correlation between CPP and outcome has been identified in non-traumatic brain injury (31).

Reduction of ICP—and thus increased CPP—may be accomplished by reducing intracranial fluid volume using agents such as the osmotic diuretic Mannitol or hypertonic saline to reduce cerebral oedema. In addition, maintaining the head elevated to 30° and in the midline facilitates cerebral venous drainage. Prevention of hypercarbia and hypoxia reduces intracerebral blood volume. All these measures may help to control ICP and therefore maintain CBF and reduce the risk of cerebral herniation and brain-stem death. However, there is no robust clinical trial data that suggests these measures improve outcome.

Signs of raised ICP include declining level of consciousness, focal neurological signs including unequal, dilated or poorly responsive pupils, systemic arterial hypertension, and associated bradycardia. Papilloedema is a late finding and is not uniformly present in acutely raised ICP.

Patients with shock without meningitis may present with impaired consciousness as a result of acidosis or cerebral hypoperfusion due to impaired cerebral perfusion due to shock. Conversely, patients without shock with meningitis may have reduced consciousness and peripheral vasoconstriction due to impaired cerebral perfusion due to raised ICP. These signs may cause confusion, with patients being misdiagnosed with shock. In this case, poor peripheral perfusion in the absence of metabolic acidosis and with normal blood lactate, together with relative bradycardia, normal, or high blood pressure and decreased level of consciousness or other neurological signs should be assumed to be due to raised ICP. If raised ICP is suspected, aggressive fluid resuscitation should be avoided, as excess fluid may exacerbate cerebral oedema.

If raised ICP is suspected, an intravenous infusion of Mannitol (0.25–0.5 g/kg over 5 min), or 3% saline (3 mL/kg over 5 min), together with urgent tracheal intubation and mechanical ventilation to control the airway and breathing and regulate blood oxygen and carbon dioxide, may prevent brain-stem herniation and may be life-saving.

Following tracheal intubation, adequate sedation must be employed in order to prevent acute waves of ICP caused by agitation or coughing. Muscle relaxants should be avoided if possible as seizures may be masked. If present, seizures should be rapidly and aggressively treated to avoid any further increase in ICP due to increased cerebral metabolism. Other neuroprotective measures include avoidance of hyperthermia, hypoxia, and by maintaining the PaCO_2_ in the normal range.

In the child with raised ICP and coexistent shock, the priority is to correct the circulatory disturbance before instituting specific measures to control ICP, although attempts to limit fluid resuscitation and maintain mean arterial pressure and blood gases in the normal range should be made to protect brain perfusion.

### Antibiotic therapy

The optimal initial antimicrobial therapy in patients with a clinical diagnosis of IMD is intravenous Ceftriaxone (100 mg/kg) but Cefotaxime (80 mg/kg) is a reasonable alternative if Ceftriaxone is contraindicated. Until definitive microbiological confirmation is available, it remains possible that alternative bacterial diagnoses or penicillin resistance (although this is extremely rare for the meningococcus) may be present. Other bacterial causes of Purpura Fulminans include *Streptococcus pneumoniae*, invasive Group A Streptococcus, *Staphylococcus aureus* and some Gram-negative bacteria. Broader antimicrobial cover may be necessary depending on the local bacterial resistance patterns and history of foreign travel or other risk factors.

The recommended duration of parenteral antibiotic therapy for uncomplicated IMD is 7 days (10).

### Adjunctive therapies

High dose corticosteroids given prior to, or concurrently with the first dose of antibiotics, appear to reduce incidence of neurological sequelae following Haemophilus influenzae type b (Hib) and pneumococcal meningitis. In meningococcal meningitis there is a trend toward improved outcome (32). The current recommendation is to administer Dexamethasone 0.15 mg/kg 6 hourly for 4 days if started before or within 4 h of the initial dose of parenteral antibiotic in patients with proven or suspected bacterial meningitis (10).

### Outcome

It is estimated that around 30% of survivors of IMD will have significant morbidity, including digital or limb amputation, skin loss or scarring, orthopedic abnormalities including abnormal bone growth, nerve deafness unilateral or bilateral, other neurological abnormalities including hemiplegia, neurodevelopmental delay, and epilepsy. Aside from these physical sequelae, a large number of affected children and their families may suffer significant neuropsychological sequelae, including post-traumatic stress disorder, depression, psychosis, reduced educational performance, and major anxiety (9, 12, 33).

#### Skin scarring

Skin scarring is particularly associated with meningococcal septicaemia. Buysse et al. reported that nearly half of children with meningococcal septicaemia had skin scarring, mainly on their extremities, face, and the trunk. Of these, 33% underwent debridement and skin grafting because of skin necrosis resulting from purpura (34).

#### Amputations/limb length discrepancies

Amputations are a consequence of meningococcal septicaemia and purpura fulminans. The prevalence of distal amputations among 515 survivors of IMD in The Netherlands was reported as 0.8% (35). Amputation appeared more common in a study from Canada, where 3.1% of 471 survivors of IMD due to serogroups B and C had amputations, which was noted to be more frequent due to serogroup C infections (36). Among Dutch childhood survivors of meningococcal septic shock, 8% had amputations of extremities, ranging from one toe to both legs and one arm (34).

Meningococcal septicaemia can also cause bone growth abnormalities, due to damage to growth plates. Of 122 Australian survivors of meningococcal septicaemia 16 patients had partial or complete physeal growth arrest (37). Limb-length discrepancies were identified in 6% of the 120 Dutch meningococcal septic shock survivors (34).

#### Other sequelae

Many studies have described the incidence and severity of varying long-term sequelae associated with IMD. One report suggests median risk of at least one sequela (major or minor, present after hospital discharge) associated with IMD, of 9.5% (95% CI: 5.1–15.1%) based on a meta-analysis of 27 studies that included 18,183 survivors of bacterial meningitis (9). The most common long-term consequences following meningococcal meningitis specifically were hearing loss (4.6%), cognitive difficulties (2.9%), and visual disturbances (2.7%). The risk of major sequelae was higher in lower-income countries, indicating the importance of good medical care in reducing long-term consequences.

#### Neurological deficits

The occurrence of long-term neurological sequelae is more common in children who have experienced more severe infections. Thirty-five percent of 120 children who survived meningococcal septic shock suffered neurological impairments on follow up 4–10 years after PICU discharge (34).

Typically, hearing loss is identified in 2–4% of IMD survivors (36, 38), including those who suffered meningococcal septic shock, not only those with meningitis. This same Dutch study identified severe mental retardation (total IQ < 70) in 3 of 120 patients; these patients also had epilepsy and two had spastic quadriplegia after meningococcal septicaemia (34).

##### Cognitive impairment and behavioral disturbances.

Several studies have identified milder neurodevelopmental deficits following IMD. A case–control study in the UK evaluated 115 child survivors of IMD ~10 years after disease onset (39). Most of these children did not have gross neurological deficits and most attended mainstream school. However, using detailed psychometric testing, survivors of IMD showed significantly lower scores compared with controls in cognition and educational performance. Educational problems are reported in >30% of children in terms of school achievement or concentration (40).

Cognitive impairments may also become apparent in older survivors of IMD. For example, a cohort of 101 adolescent IMD survivors (aged 15–19 years at time of disease) in the UK followed-up over a period of 1.5–3 years showed poorer educational outcomes compared with controls (41).

##### Psychological problems.

Several studies have reported that IMD can lead to delayed psychological problems such as post-traumatic stress disorder (PTSD), anxiety and depression. A UK study in 56 children (aged 3–16 years) indicated that there were statistically significant increases in emotional distress, hyperactivity and attention deficit disorder and at 12 months after discharge 11% of the children were considered at risk for PTSD (33).

Experience of IMD has significant psychological consequences for the parents of the index child. In interviews carried out 3–12 months following admission, nearly half the mothers of children with IMD were considered to be at psychiatric risk with the possibility of developing PTSD (42) and nearly a third of the other mothers were seeking professional help for psychological difficulties.

## Prevention of meningococcal disease

Invasive meningococcal disease is of great concern to physicians whenever assessing a child with a febrile illness. In its early stages, it often presents with non-specific symptoms and is therefore very difficult to diagnose early. If misdiagnosed, the disease can progress rapidly with the patient dying within 24–48 h. Because of these factors and the fact that most disease occurs in young children, effective immunization of vulnerable populations is desirable.

There are two main classes of vaccine used for protection against IMD: pure polysaccharide vaccines and protein/polysaccharide conjugate vaccines. Both of these types of vaccines are based on the capsular polysaccharide of the meningococcus, which is a major virulence factor and is primarily responsible for colonization and invasion by prevention of host-mediated bacterial killing.

The host immune response to pure polysaccharide vaccines is not efficient in young children under the age of 2 years, because of reduced immune responsiveness to T-cell-independent antigens such as polysaccharides. This is an important limitation of vaccines based on these structures given that the highest risk for acquisition of IMD is in children < 5 years of age. In addition, pure polysaccharide vaccines do not induce mucosal immunity and thus fail to prevent nasopharyngeal carriage of meningococci, and therefore cannot influence herd immunity.

In summary, pure polysaccharide vaccines are poorly immunogenic in children under 2 years of age, and confer inadequate and only temporary immunity, lasting for ~3–5 years in older children and adults. They do not impact nasopharyngeal carriage of pathogenic meningococci and in recent years they have been superceded by the conjugate protein/polysaccharide vaccines (32).

In recent years, effective quadrivalent conjugate polysaccharide vaccines have become available against the meningococcal serogroups A, C, Y, and W.

As MenA was the primary cause of IMD in the meningitis belt of sub-Saharan Africa, and was responsible for thousands of epidemic cases and deaths each year, the WHO initiated the Meningitis Vaccine Project, which developed a low-cost conjugate vaccine against MenA (MenAfriVac). This vaccine was successfully launched in mass vaccination campaigns as a single dose in over 250 million people aged 1–29 years across 25 countries in the African meningitis belt (4). Following this, a major reduction in numbers of cases of MenA disease in sub-Saharan Africa was observed.

MenC conjugate vaccine has been successfully introduced into much of Europe, and is now part of the routine immunization schedule. This vaccine is strongly immunogenic, giving relatively long-lasting immunity and immunological memory, and it also has a significant impact on decreasing nasopharyngeal carriage in vaccines and therefore confers herd immunity or community protection.

Since the introduction of MenC conjugate vaccine into the UK infant schedule in 1999, the incidence of MenC disease has decreased by 94% in immunized populations and 67% in unimmunized populations due to herd immunity (43). There has been a demonstrated important decrease in nasopharyngeal carriage, with, importantly, no increase in the carriage of other disease causing serogroups (44). In the epidemiological year 2016–2017 in all ages in the UK, there were 37 cases of MenC disease in total (6). Because of these low numbers of reported cases of MenC (and the ongoing vaccination programme with MenACWY quadrivalent conjugate vaccine in teenagers), in 2018 all infant Men C vaccine doses have been removed from the UK schedule, leaving a priming dose at 1 year and a booster in adolescence (as part of the MenACWY vaccine).

### Vaccines for serogroup B meningococcus

Following the successful introduction of MenC vaccine, MenB has remained the most common cause of IMD, accounting for more than 50% of cases in the USA and as many as 90% of cases in Europe (5).

MenB has a poorly immunogenic capsule due to its polysaccharide structure. The polysaccharide capsule of MenB is composed of polysialic acid (α2–8 N-acetylneuraminic acid) which has structural similarity to carbohydrates found in fetal brain tissue. Therefore, there is a degree of immune tolerance to this polysaccharide, and there are concerns regarding the effect of modifying the sugar structure in a vaccine to make it more immunogenic, in case of induction of auto-immunity. These factors have therefore hindered progress on developing an effective polysaccharide vaccine as this molecular similarity does not allow the development of an effective protein/polysaccharide conjugate vaccine for MenB (45).

Because of these factors, vaccine development for MenB has required a different approach. MenB vaccines have now been developed targeting non-capsular structures, such as outer membrane porins, vesicles, and lipopolysaccharide (LPS).

LPS is a universal component of the outer membrane of Gram-negative bacteria and contributes to pathogenicity of the meningococcus. *Neisseria meningitidis* also produces various surface proteins, one of which, factor H binding protein, fHbp, binds human complement factor H. Human Factor H is important in regulating the alternative complement pathway. Binding of human Factor H to the surface of meningococci by bacterial fHbp is thought to inhibit complement-mediated bacterial lysis. Variations in human Factor H and related proteins have been found to be important factors in determining susceptibility to meningococcal disease (46, 47).

Another meningococcal surface protein, Neisserial Surface Protein A (NspA), can also bind human complement Factor H (48). Other factors which mimic or bind host molecules also inhibit complement-mediated bacterial lysis and phagocytosis. For example, N meningitidis sheds blebs of outer membrane which contain these outer membrane proteins and LPS. These outer membrane vesicles (OMVs) have been shown to initiate complement activation and might redirect complement activation away from invading meningococci in the circulation, thereby hindering the bactericidal effects of human complement.

In order to try to develop a universal vaccine against MenB, researchers at Novartis Vaccines used the genomic sequence of a serogroup B strain (N meningitidis serogroup B strain MC58)—a process called “reverse vaccinology” (49). This eventually led to development of an immunogenic MenB vaccine with novel vaccine protein antigens (a 4 component vaccine).

This novel 4CmenB (Bexsero) vaccine was introduced into the UK infant schedule in September 2015. Latest figures estimate a vaccine uptake of 92.6% for the 2 infant doses by 52 weeks of age. There has been a definite impact on incidence of MenB in infants, in 2015/16 there was an incidence of 17 per 100,000 which was reduced to 11/100,000, a fall of 11% overall, following vaccine introduction (6). It is unknown whether this vaccine will have any effect on nasopharyngeal carriage and therefore provide herd protection. Even if 4CMenB had a definite effect on carriage, herd immunity will not occur whilst only infants are vaccinated as this will have no impact on the main source of carriage, which are adolescents.

Another vaccine effective against MenB, manufactured by Pfizer, which contains antigenic components from subfamilies A and B of meningococcal fHbp has undergone extensive clinical evaluation in young adults and adolescents. This vaccine (Trumemba) has been widely used in North America in outbreaks of MenB disease in universities and has been shown to be safe and effective (50).

Studies in vaccinees who have received either Bexsero or Trumemba are being carried out in the UK to establish whether either or both of these vaccines have any effect on nasopharyngeal carriage, a prerequisite for the establishment of herd immunity. However, the only way to truly assess the effects of these vaccines on disease incidence and indirect protection is to carry out enhanced surveillance following population-wide vaccine introduction. It is important to consider the possibility that any protein-based MenB vaccine would have negligible or no impact on nasopharyngeal carriage, in which case there would be no clear strategy to induce herd immunity. This may suggest that the only way to reduce MenB incidence would be widespread all age immunization which may be economically unviable. However, if an impact on nasopharngeal carriage is demonstrated, then strategies to provide herd immunity, such as immunization of adolescents or adults could be appropriate.

Another potential benefit of use if these sub-capsular outer-membrane protein vaccines is their potential to induce cross protection against other meningococcal strains because the components of these vaccines are highly conserved protein antigens present in a high proportion of meningococcal subtypes irrespective of their covering capsule. This raises the possibility of development of a universal meningococcal vaccine, potentially leading to eradication of meningococcal disease (51).

## Conclusion

The impact of conjugated meningococcal vaccines on disease incidence has been enormous. Countless young lives have been saved or dramatically improved due to disease prevention.

The challenge now is to continue to be vigilant in those populations that remain unimmunized so that appropriate recognition and management of invasive meningococcal disease is prioritized. In the meantime, it is clear that following guidelines and implementation of treatment bundles can have a significant effect on outcome.

We await data regarding the effectiveness of the newer protein vaccines against serogroup B meningoocccus on carriage and their cross-reactivity against non-B serogroups of meningococcus.

We look forward to a world without meningitis and septicaemia.

## Disclosure

SN has received consulting fees from Novartis and Pfizer. NN has received consulting fees from GSK and Pfizer.

## Author contributions

All authors listed have made a substantial, direct and intellectual contribution to the work, and approved it for publication.

### Conflict of interest statement

The authors declare that the research was conducted in the absence of any commercial or financial relationships that could be construed as a potential conflict of interest.
